# Effects of a virtual reality serious game training program on the cognitive function of people diagnosed with schizophrenia: A randomized controlled trial

**DOI:** 10.3389/fpsyt.2022.952828

**Published:** 2022-07-15

**Authors:** Xu Wang, Xiaomin Kou, Xiandong Meng, Jianying Yu

**Affiliations:** ^1^West China Hospital, Sichuan University, Chengdu, China; ^2^West China School of Nursing, Sichuan University, Chengdu, China

**Keywords:** schizophrenia, virtual reality, serious game, cognitive function, randomized controlled trial

## Abstract

**Background:**

Cognitive impairment persists through the course of schizophrenia and affects patients’ activities of daily living.

**Aim:**

This study aims to investigate the effects of a virtual reality (VR) serious game training program on the cognitive function of people diagnosed with schizophrenia.

**Materials and Methods:**

Sixty-four eligible people diagnosed with schizophrenia were recruited and randomly assigned to the intervention group (*n* = 31) and the control group (*n* = 33). The control group received standard psychiatric care. The intervention group was trained with an additional VR game twice a day for at least 10 days during hospitalization. Cognitive function was measured at enrollment and before discharge using the Brief Cognitive Assessment Tool for Schizophrenia.

**Results:**

Compared with those of the control group, the results of the working memory (*t* = 3.463, Cohen’s d = 0.87, *p* = 0.001) and executive function (TMTA: Z = -2.272, Cohen’s d = 0.59, *p* = 0.023; TMTB:Z = -2.365, Cohen’s d = 0.62, *p* = 0.018) of the intervention group after intervention were significantly better. However, there was no significant difference in the results of social cognition (*Z* = -1.394, Cohen’s d = 0.35, *p* = 0.163) between the two groups.

**Conclusion:**

Intensive active virtual reality serious game training in addition to standard psychiatric care can significantly improve working memory and executive function in people diagnosed with schizophrenia.

**Implications for Practice:**

When helping improve the cognitive function of people diagnosed with schizophrenia, mental health professionals should identify cognitive domains to be enhanced and develop corresponding serious game training strategies.

## Introduction

Schizophrenia is a complex serious mental disorder and one of the top 10 disabling diseases, affecting 21 million people in the world (1). It is usually noticed and diagnosed by positive symptoms, in particular delusions and hallucinations (2). Negative symptoms, such as diminished emotional expression, anhedonia and asociality, have also been included in the diagnostic criteria of schizophrenia as one of the five core dimensions in the Diagnostic and Statistical Manual of Mental Disorders 5th edition (DSM-5) (3). In recent years, some psychiatrists have proposed that focusing solely on psychotic symptoms of schizophrenia may be a conceptual fallacy and hampers progress in understanding and treating the disorder (4, 5).

Schizophrenia is associated with impairment across a wide range of higher-order cognitive performance domains (6). Cognitive impairment has been detected even before the apparent onset of schizophrenia, persists throughout the course of the disease and is further aggravated during acute episodes (7). Approximately 75 to 80% of people diagnosed with schizophrenia have cognitive impairment, including a decline in attention test, processing speed, visual and verbal learning, working memory and executive function, which makes it difficult for patients to accurately perceive, process, and remember information (8). Consequently, people’s ability to study, work or manage daily affairs is impaired, which in turn leads to reduced social function (9). Studies have suggested that, compared with psychotic symptoms, cognitive function is more strongly correlated with and predictive of functional outcomes of people diagnosed with schizophrenia (10, 11). Therefore, maintaining or even enhancing cognitive function is of great significance in improving the prognosis of people diagnosed with schizophrenia.

However, the results of interventions for cognitive impairment in people diagnosed with schizophrenia have been mixed. Antipsychotics are the frontline treatments for schizophrenia and are effective for psychotic symptoms, but their effect on cognitive impairment is neutral (12, 13). In addition, metabolic side effects caused by antipsychotics, especially second-generation antipsychotics, may lead to further deterioration of cognitive impairment (14). Other pharmacological approaches, such as cognitive enhancement targeting various neurotransmitter systems, produced small effects on global cognition and no significant effects on any subdomains of cognitive function (15).

On the contrary, fairly intensive cognitive training programs have had promising findings. Wykes et al. (16) systematically reviewed 40 studies of cognitive training programs with 2104 participants and found that such programs produced a small to moderate effect of cognitive improvement in people diagnosed with schizophrenia despite the treatment duration and training modality, and these benefits were not compromised by poor study methods. Of those, cognitive training programs involving teaching strategies used in the tasks or combined with psychiatric rehabilitation had a much larger effect. Another intervention with a positive cognitive outcome was physical exercise. A recent meta-analysis demonstrated that aerobic exercise significantly improved the global cognition of people diagnosed with schizophrenia (17).

Despite the encouraging results of non-pharmacological interventions, one common problem that hinders cognitive function recovery is participant’s low involvement or high dropout rate. Affected by negative symptoms of schizophrenia and the sedative effect of antipsychotics, people diagnosed with schizophrenia are less motivated to participate in treatments and more likely to drop out of treatments halfway. To maintain and increase participant’s involvement, interventions need to be interesting and attractive. Serious games can fulfill this requirement. Serious games are mental contests designed to mimic specific activities that utilize entertainment to educate or train people in a virtual environment (18). What differentiates serious games from entertainment games is that their development requires the formulation of rules and techniques based on certain therapeutic theories. For example, guided by cognitive load theory, a titrated increase in difficulty helps participants maintain focus during the game (19); in order to change biased perceptions of social interaction, the techniques of cognitive behavioral therapy need to be incorporated into gameplay (20). There has been a growing amount of research using serious games to amplify learning and motivation in the therapeutic prevention and treatment of patients with various medical conditions (21, 22).

To remediate the cognitive function of people diagnosed with schizophrenia, we developed a virtual reality serious game based on the theory of neuroplasticity, which advocated that the brain nervous system can constantly adjust the connection between nerves under the stimulation of the external environment, forming functional reorganization (23). The program had several advantages for the enhancement of cognitive function, including a simple immersive interactive environment, progressive increase in difficulty, motivational competition and physical movements. The hypothesis of this study was that an intensive cognitive training program with an active virtual reality (VR) serious game can improve cognitive function in people diagnosed with schizophrenia. The results are now reported as follows.

## Materials and methods

### Study design and participants

This was a single-center randomized controlled trial conducted in Chengdu, Sichuan Province, China, from June 2020 to May 2021. Participants were recruited from inpatients at the mental health center of a tertiary general hospital. Clinical nurses referred potential participants to the study in routine medical service. The researchers of this study screened all referrals for eligibility. Eligible participants were adults between the ages of 18 and 40, diagnosed with schizophrenia by an experienced psychiatrist according to the International Classification of Disease-10 (ICD-10), without a history of head trauma or substance abuse or neurological diseases affecting cognitive function, and could understand and speak Mandarin. This was the first time that many patients had used VR equipment. Therefore, any patient diagnosed with schizophrenia who would like to enroll in the program underwent an experimental session. Exclusion criteria were diagnosis of color blindness, feeling negative effects such as dizziness and fatigue after using VR equipment, failing to complete 20 training sessions, participating in other physical therapy programs to improve cognitive function, or being unable to sign informed consent.

PASS 15.0 software was used to calculate the sample size. The effect size was set at Cohen’s d = 0.8 based on previous studies (16, 24). The two-sided test efficacy was 0.05, and the power was 0.80. Thus, the sample size was 50. In the case of a 20%loss to follow-up, 64 participants were recruited according to the inclusion and exclusion criteria.

### Randomization and blinding

Sixty-four random numbers were generated by a research assistant who did not participate in the recruitment and intervention of the participants using an online random number generator on a website. Each random number corresponded to a number from 1 to 64 without repetition, forming a random number sequence, which was recorded and sealed in opaque envelopes. The even number was set as the intervention group, and the singular number was set as the control group. Participants were given numbered envelopes in the order in which they were enrolled and then randomly assigned to either the intervention group or the control group.

The psychiatric nurse who organized and carried out participants’ mental rehabilitation activities knew the allocation of participants. Participants were informed of the activities they might participate in, but they did not know whether they were in the intervention group or the control group. Researchers who collected demographic data and assessed the outcome measures were blinded to allocation and had no access to records of participants’ mental rehabilitation activities to avoid unmasking. Since the VR serious game program was used as an adjunctive intervention in this study, all participants still followed the psychiatrist’s advice for medication during the study. To minimize differences in medication regimens while not interfering with participants’ routine treatment, all participants were selected from patients with the same psychiatrist. To avoid possible objective bias, the psychiatrist was blinded to the participants’ allocation.

### Procedures

**FIGURE 1:**
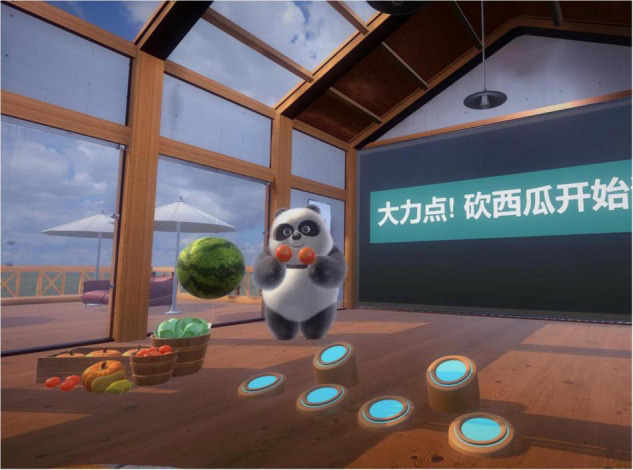
Users’ field of vision from virtual reality (VR) Google.

**FIGURE 2:**
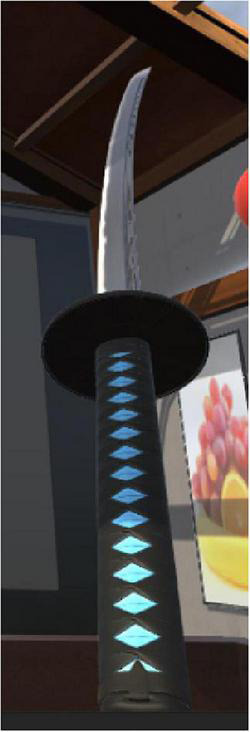
The long knife was controlled by a hand device.

“Fruit Pioneer” was an active VR serious game adapted from popular fruit-cutting games. People diagnosed with schizophrenia generally had impaired cognitive function, reduced ability to deal with complex situations and reduced attention. Fruit Pioneer reduced the interference of external sound and pictures by using a VR head-mounted display and headphones. The software was developed using Unity3D (version 2018.3.0f2, Unity Technologies) and ran on a personal computer (HP PC, Intel Core i5-9400F processor, 16 GB DDR4 3000 MHz memory, GTX 1660 6GB graphics card, 256 GB solid state drive and 1 TB hard disk drive) and the HTC Vive (HTC Corporation) VR head-mounted display. The game was played from a first-person perspective, with a swinging panda, a scoreboard and a circle of fruit-shooting spouts in the users’ field of vision (see Figure 1). Headphones of the VR googles played joyful game sounds that changed with the content and pace of the game. Users held a handle device in each hand, and the device was instantly connected to the VR googles when pressed its bottom, which visually changed into two long knives (see Figure 2). Users were given the task of cutting as many fruits as possible while avoiding iron balls. Fruits may be shot from any of the spouts. Therefore, users were expected to turn around and search for fruits. The fruits were yellow bananas, red apples, and green watermelons that were common in daily life and varied in color, shape, and size. The smaller the fruit, the more points it would earn after successfully cutting it. Cutting to the iron ball did not penalize points but created turbulence that interfered with the next cut. Each level of the game was 2 min long. Shooting speed, direction and numbers of fruits can be adjusted to the level of difficulty. The further the game went, the more difficult it got. That is, the faster and more scattered the fruit appeared, the more likely the iron ball and fruit appeared at the same time.

**FIGURE 3:**
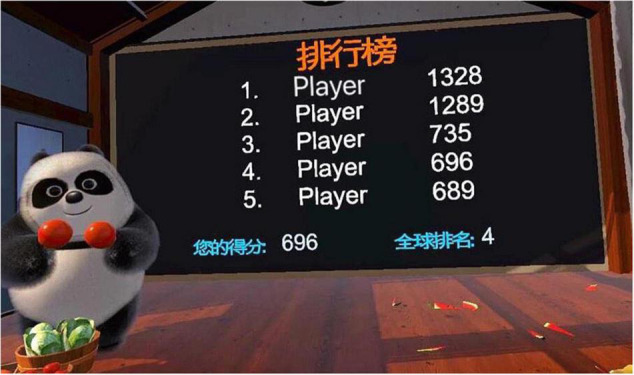
The scoreboard displayed the participant’s score and ranking.

Participants in the intervention group were guided to the physical therapy room for VR serious game training by a psychiatric nurse. The psychiatric nurse explained game rules and strategies involved in the Fruit Pioneer and accompanied participants in case of any adverse event. The game training was one-on-one. To ensure the safety of participants and reduce the sense of insecurity caused by the virtual environment, their activities were confined and protected by a plastic fence with a diameter of 2 m. Each level of the game lasted 2 min. After completing one level, there was a 10-second pause, and the scoreboard displayed the participant’s score and ranking for the level and the highest score of the level created by other anonymous participants (see Figure 3). Participants then chose to repeat the level or start the next level. Each training session lasted 20 to 30 min, twice a day. The psychiatric nurse recorded the participant’s training sessions. The total number of training sessions was at least 20 times for each participant during hospitalization. This training program was given to participants in addition to standard care.

Participants in the control group received standard and routine inpatient psychiatric care, including medication, psychotherapy and group psychiatric rehabilitation. Group psychiatric rehabilitation activities were organized and guided by psychiatric nurses in the rehabilitation room, which was located in a different area from the physical therapy room. Psychiatric rehabilitation activities were carried out around the daily life, study, work and social interaction of people diagnosed with schizophrenia, including psychoeducation about symptom and medication management, learning or occupational goal setting and plan making, and social skills training. Such activities lasted for approximately 45 min, once a day. Participants in the intervention group could also participate in these activities if they wanted to.

The arrangement of psychiatric treatment and nursing care was based on participants’ actual medical needs. During hospitalization, each participant was checked for content and times of participation in psychotherapy and psychiatric rehabilitation activities according to prescription in the electronic medical record system to ensure that there was no difference between the two groups except for the VR serious game training. All enrolled participants had demographic data collected by researchers and completed an initial B-CATS cognitive assessment upon enrollment. Then, B-CATS was evaluated again before discharge. All researchers received the same training on study content, methods and assessment tools prior to the study.

### Outcomes

A general demographic questionnaire was used to collect gender, age, education background, and marital status.

Primary outcomes were scores of the Brief Cognitive Assessment Tool for Schizophrenia (B-CATS). The B-CATS is a simple instrument to measure approximate cognitive function that generally takes 12 min to finish. It was constructed by Hurford et al. (25) and contained 4 tests, namely, the Digital Symbol Substitution Test (DSST), Trail Making Test part A (TMTA), Trail Making Test part B (TMTB), and Animal Fluency (AF). In the DSST, participants were presented with a sheet having 9 symbols paired with digits 1–9 on the top and rows of symbols beneath. Participants were asked to pair each symbol with its corresponding digit within 120 s. The number of correct pairs was the score. There were 120 symbols on the sheet, the first 10 of which were used for examples. Therefore, the maximum score for this test was 110. The DSST reflected the participant’s work memory. TMTA and TMTB demanded that participants draw a “trail” in numerical order of numbers or from number to letter (1-A-2-B) without taking the pen off the sheet. Participants were stopped and returned to the last correct response when they made a mistake. Time to completion was the score. TMT indicated the participant’s executive function. In the AF part, participants were given 60 s to name as many animals as possible. The score was the number of animals named. AF measures verbal fluency associated with social cognition. The B-CATS presented good divergent validity, test-rest reliability and internal consistency (25). In addition, it can be easily administered by medical staff with proper training, suitable for use in this study.

### Ethical consideration

This study was ethically approved by the Biomedical Ethics Committee of West China Hospital, Sichuan University (reference: 2019-468). All methods were carried out in accordance with relevant guidelines and regulations.(e.g., Helsinki declaration). The procedures and content of the study were explained to all participants. Participants were informed that they could withdraw from the study at any time, and it would not interfere with their medical services. Written informed consent was obtained before any interventions. This trial is registered with the Chinese Clinical Trial Registry, number ChiCTR1900028041.

### Data analysis

EpiData3.0 software was used to input and manage demographic data and neurocognitive function assessment data. SPSS 21.0 statistical analysis software was used for data analysis. *P* < 0.05 indicates that the difference is statistically significant. Count data were described by frequency and composition ratio and compared by the Fisher’s exact test. Measurement data with a normal distribution were described by the mean plus or minus the standard deviation, and measurement data with a non-normal distribution were described by the median and first quartile and third quartile. The Shapiro–Wilk test was used for normality testing. An independent *t* test was used to compare the data with a normal distribution. If the data did not obey a normal distribution, the Mann–Whitney U test was used for comparison. To ensure the robustness of the findings, independent *t* test was used for sensitivity analysis after log-transformation of skewed outcomes. Analyzes were done by complete case analysis.

## Results

### Comparison of demographic characteristics

**FIGURE 4:**
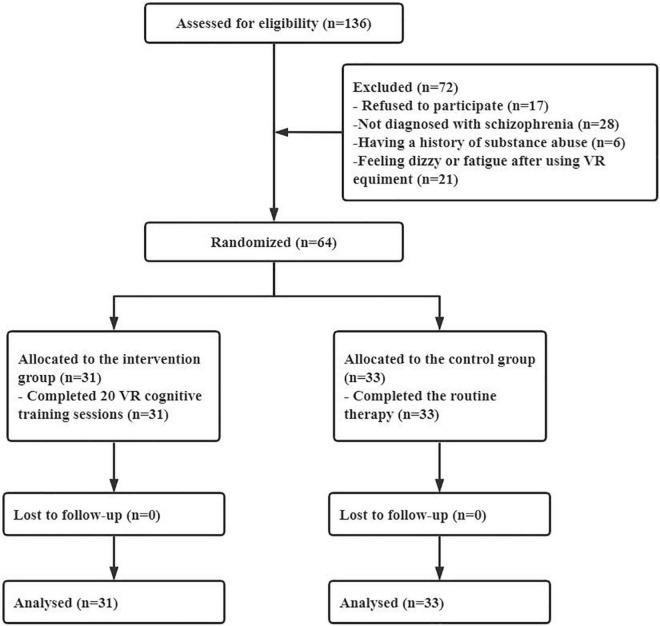
Flow diagram of the study.

In this study, 64 participants were finally included and randomly assigned to the intervention group (*n* = 31) and the control group (*n* = 33) (see Figure 4). No participants withdrew midway or failed to complete 20 sessions of training. The average VR serious game training sessions in the intervention group were 25.84 (standard deviation = 4.03). The demographic data of the participants in the two groups were compared. There was no significant difference in sex, age, length of hospital stay, education background or marital status between the intervention group and the control group, as shown in Table 1.

**TABLE 1:** Comparison of demographic characteristics between the intervention group and the control group.

Variables		(M ± SD)/%		t(df)/χ *2*	*P*-value
		Intervention group (*n* = 31)	Control group (*n* = 33)		
Gender	Male	14 (45.2%)	17 (51.5%)	0.259	0.627
	Female	17 (54.8%)	16 (48.5%)		
Age (year)		24.61 ± 5.68	26.94 ± 6.27	-1.552	0.126
Length of hospital stay (day)		17.42 ± 4.63	18.03 ± 6.88	-0.414	0.680
Education background	Primary school	0 (0%)	2 (6.1%)	3.229	0.760
	Junior high school	3 (9.7%)	4 (12.1%)		
	Senior high school	6 (19.4%)	7 (21.2%)		
	Junior college	13 (41.9%)	10 (30.3%)		
	Undergraduate	9 (29.0%)	9 (27.3%)		
	Graduate	0 (0%)	1 (3.0%)		
Marital status	Unmarried	21 (67.8%)	19 (57.6%)	0.888	0.626
	Married	9 (29.0%)	12 (36.4%)		
	Divorced	1 (3.2%)	2 (6.0%)		

### Baseline cognitive function

Before comparing the baseline cognitive function of the intervention group and the control group, the Shapiro–Wilk test was performed on B-CATS scores. The results showed that only the DSST scores obeyed a normal distribution (*p* = 0.836), while the TMTA (*p* < 0.001), TMTB (*p* < 0.001), and AF (*p* = 0.013) scores did not. Therefore, an independent *t* test was used to compare the DSST, and the Mann–Whitney U test was used to compare the TMTA, TMTB, and AF between the two groups. There were no significant differences between the two groups in the baseline scores of the DSST, TMTA, TMTB, and AF. The results are shown in Table 2.

**TABLE 2:** Comparison of baseline cognitive function between the intervention group and the control group.

Variables	(M ± SD)/[M(Q1,Q3)]		*t/Z*	*P*-value
	Intervention group (*n* = 31)	Control group (*n* = 33)		
DSST^a^	52.97 ± 14.84	43.30 ± 14.28	2.656	0.880
TMTA^b^	41.00 (30.00, 51.00)	45.00 (31.00, 62.50)	−0.820	0.412
TMTB^b^	92.00 (63.00, 118.00)	92.00 (76.00, 180.50)	−1.673	0.094
AF^b^	19.00 (18.00, 21.00)	18.00 (14.00, 22.50)	−0.936	0.349

(M ± SD), (mean ± standard deviation); [M(Q1-Q3)], [median(first quartile, third quartile)]; DSST, digital symbol substitution test; TMTA, trail making test part A; TMTB, trail making test part B; AF, animal fluency.

^a^Means that the difference was assessed by independent *t* test.

^b^Means that the difference was assessed by Mann–Whitney U test.

### Comparison of cognitive function after intervention

**FIGURE 5:**
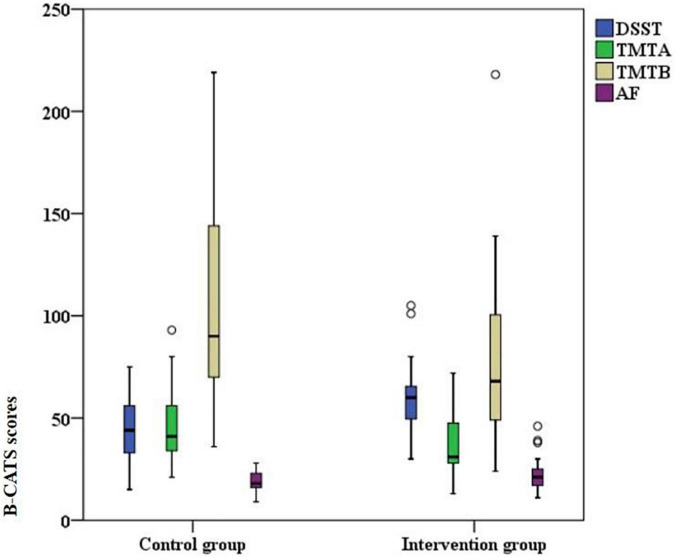
Scores on the four Brief Cognitive Assessment Tool for Schizophrenia (B-CATS) tests after intervention.

**FIGURE 6:**
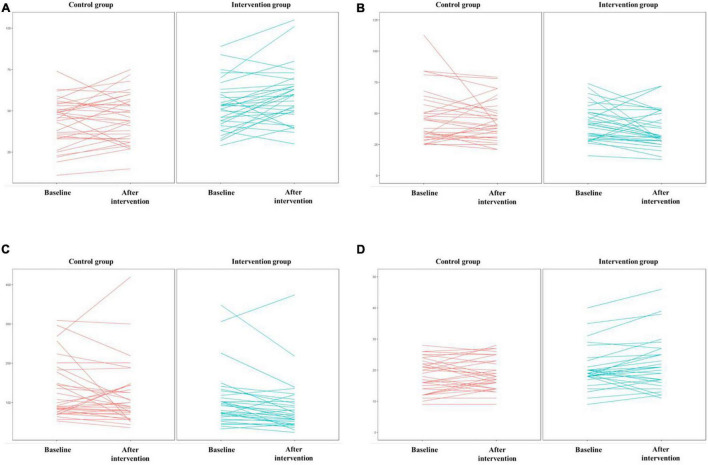
Scores on the four Brief Cognitive Assessment Tool for Schizophrenia (B-CATS) tests. **(A)** DSST, **(B)** TMTA, **(C)** TMTB, **(D)** AF.

The results of the Shapiro–Wilk test showed that the DSST scores obeyed a normal distribution (*p* = 0.110), while the TMTA (*p* = 0.001), TMTB (*p* < 0.001), and AF (*p* < 0.001) scores did not. Thus, an independent *t* test was used to compare the DSST, and the Mann–Whitney U test was used to compare the TMTA, TMTB, and AF between the two groups. The DSST scores of participants in the intervention group were significantly higher than those in the control group. Participants in the intervention group completed TMTA and TMTB significantly faster than those in the control group. There was no significant difference in AF scores between the two groups. Details of scores were shown in Table 3. Distribution of outcomes after intervention were shown in Figure 5 and changes of B-CATS scores from baseline were shown in Figure 6.

**TABLE 3:** Comparison of cognitive function between the intervention group and the control group after intervention.

Variables	(M ± SD)/[M(Q1,Q3)]		*t/Z*	*P*-value	Effect size (Cohen’s d)
	Intervention group (*n* = 31)	Control group (*n* = 33)			
DSST^a^	59.19 ± 16.78	45.64 ± 14.51	3.463	0.001	0.87
TMTA^b^	31.00 (28.00, 50.00)	41.00 (32.50, 59.00)	−2.272	0.023	0.59
TMTB^b^	68.00 (46.00, 103.00)	90.00 (70.00, 144.50)	−2.365	0.018	0.62
AF^b^	21.00 (17.00, 25.00)	18.00 (15.00, 23.00)	−1.394	0.163	0.35

(M ± SD), (mean ± standard deviation); [M(Q1-Q3)], [median(first quartile, third quartile)]; DSST, digital symbol substitution test; TMTA, trail making test part A; TMTB, trail making test part B; AF, animal fluency.

^a^Means that the difference was assessed by independent *t* test.

^b^Means that the difference was assessed by Mann–Whitney U test.

Scores of TMTA, TMTB, and AF were log-transformed and compared with independent *t* test to ensure the robustness of the findings. Results were the same that was, the scores of TMTA and TMTB in the intervention group were significantly lower than those of the control group, and there was no significant difference in the AF scores between the two groups. Results were shown in Table 4.

**TABLE 4:** Sensitivity analysis using independent t test for log-transformed skewed outcomes.

Variables	(M ± SD)		*t*	*P*-value	Effect size (Cohen’s d)
	Intervention group (*n* = 31)	Control group (*n* = 33)			
TMTA	3.52 ± 0.40	3.76 ± 0.39	−2.522	0.014	0.61
TMTB	4.28 ± 0.57	4.61 ± 0.55	−2.351	0.022	0.59
AF	3.02 ± 0.35	2.90 ± 0.28	1.613	0.112	0.38

(M ± SD), (mean ± standard deviation); DSST, digital symbol substitution test; TMTA, trail making test part A; TMTB, trail making test part B; AF, animal fluency.

## Discussion

There was no significant difference in the demographic data between the intervention group and the control group, indicating that the two groups were comparable. At the same time, there were no significant differences in baseline B-CATS results, suggesting that the two groups had the same degree of cognitive impairment prior to intervention. The results of this study showed that the intensive VR serious game training program significantly improved participant’s working memory and executive function, but had little effect on social cognition.

People with schizophrenia have impaired working memory, which means that their ability to temporarily store and process information is reduced (26). In this study, the DSST scores of people diagnosed with schizophrenia in the intervention group were significantly higher than those in the control group after intervention, indicating that intensive active VR serious game training improved working memory in the intervention group. This was consistent with a previous study showing that serious game training enhanced visuospatial working memory (27). There are two strategies commonly used in the DSST. One way is to look for the digit corresponding to each symbol in the example during the whole test process. The other is to memorize symbols and their corresponding digits first and then fill in the corresponding digits of symbols in the blank space. Usually, the latter strategy accomplishes more. This VR serious game used the latter strategy as the training paradigm. Before the game, participants were informed of scores of different fruits (symbols), with an emphasis on the goal of the game, namely, to get as many points as possible in a limited time. At the end of each game, participants could compare their scores with scores and rankings on the scoreboard created by other players. The smaller the difference, the more correct the participant’s strategy was. To obtain more scores, participants needed to quickly respond to the fruits with high scores and constantly optimize the strategy during the training sessions to achieve the training and improvement of working memory.

After intervention, the test time of TMTA and TMTB in the intervention group was significantly shorter than that in the control group. TMT reflects executive function by evaluating visual search, cognitive flexibility and psychomotor speed (28). The results of this study indicated that VR serious game training improved the executive function of the intervention group, which was consistent with previous studies. Rozental-Iluz et al. (29) found that interactive video game training increased motor speed and cognitive flexibility in individuals with chronic stroke. Shimizu et al. (30) demonstrated that interactive sports serious game training was correlated with increased executive function performance in people diagnosed with schizophrenia. People diagnosed with schizophrenia showed executive function deficits manifested as difficulty in making and implementing plans, solving problems, and completing target tasks (31). The cutting of fruits in this study required participants to conduct a visual search of the target fruit and then perform the cutting by quickly identifying and integrating different properties of the target. In addition, constant attempts to avoid iron balls trained inhibition. The potential underlying rationale is that repeated VR target cutting stimulates the central nervous system to generate new synaptic connections or repair existing connections, thus improving the executive function of patients, according to the theory of neuroplasticity.

There was no significant difference in AF scores between the intervention group and the control group. AF measures verbal fluency, which is closely related to social function (32). Grimes et al. (33) found that verbal fluency was stable across multiple stages of schizophrenia even with antipsychotic medications and suggested that it could be a neurocognitive endophenotype for schizophrenia, partly self-explaining its stability. Even physical exercise, which was strongly advised for cognitive improvement, had only a marginally significant effect on verbal fluency (34). In contrast, psychosocial interventions, which encouraged participants to socialize with others, improved verbal fluency (35, 36). This probably explained the ineffectiveness of the active VR serious game training in this study. Unlike psychosocial interventions, this training program barely involved any language training of participants. It was suggested that cognitive training needed to be targeted, and each subdomain required different training strategies.

This trial has some limitations. First, participants and researchers responsible for the interventions were not blinded. There was a perceptible difference in the activities between the intervention group and the control group. Although we set the activities in different rooms to reduce the direct impact, it was possible that the participants were subjectively affected. We blinded the researchers who evaluated the outcomes to decrease subjective bias. Second, the active feature of the VR serious game training (such as standing and turning around during the training) could contribute to the positive outcome of this study. However, indicators reflecting physical exercise were not monitored in this trial, so further studies are needed. Third, the sample size of this study was calculated and adequate for a randomized controlled trial, but the overall representation of cognitive function in people diagnosed with schizophrenia was limited.

## Conclusion

The results of this study showed that providing intensive active virtual reality serious game training in addition to standard psychiatric care can significantly improve working memory and executive function in people diagnosed with schizophrenia. However, global cognition enhancement requires more comprehensive serious game training strategies.

## Relevance for clinical practice

Intensive active virtual reality serious games with attention, memory and reaction speed as the main training targets can significantly improve neurocognition, including working memory and executive function. However, such a training strategy had limited influence on social cognition. These two aspects of cognitive function require different training strategies. Therefore, when mental health professionals try to design a program to improve the global cognitive function of people diagnosed with schizophrenia, they need to identify the cognitive domains to be enhanced and then develop corresponding serious game training strategies.

## Data availability statement

The raw data supporting the conclusions of this article will be made available by the authors, without undue reservation.

## Ethics statement

The studies involving human participants were reviewed and approved by Medical Ethics Committee of West China Hospital, Sichuan University. The patients/participants provided their written informed consent to participate in this study.

## Author contributions

XW, XM, and JY contributed to the conception and design of the study. XW and XK organized and implemented the intervention. XW performed the statistical analysis and wrote the first draft of the manuscript. XK, XM, and JY wrote sections of the manuscript. All authors contributed to manuscript revision, read, and approved the submitted version.

## Conflict of interest

The authors declare that the research was conducted in the absence of any commercial or financial relationships that could be construed as a potential conflict of interest. The reviewer CG and handling editor declared their shared affiliation.

## Publisher’s note

All claims expressed in this article are solely those of the authors and do not necessarily represent those of their affiliated organizations, or those of the publisher, the editors and the reviewers. Any product that may be evaluated in this article, or claim that may be made by its manufacturer, is not guaranteed or endorsed by the publisher.
